# Genotyping of respiratory syncytial virus among influenza‐like illness and severe acute respiratory infection cases of children in the Philippines from 2006 to 2016

**DOI:** 10.1111/irv.12986

**Published:** 2022-05-18

**Authors:** Jonjee Calaor‐Morin, Vina Lea Arguelles, Janiza Lianne Foronda, Alvin Tan, Evelina Lagamayo, Clyde Dapat, Socorro Lupisan

**Affiliations:** ^1^ Department of Virology Research Institute for Tropical Medicine Muntinlupa Philippines; ^2^ Graduate School University of Santo Tomas Manila Philippines; ^3^ Department of Epidemiology and Biostatistics Research Institute for Tropical Medicine Philippines; ^4^ Department of Medicine University of Santo Tomas Manila Philippines; ^5^ RITM‐Tohoku Collaborating Research Center on Emerging and Re‐emerging Infectious Diseases Muntinlupa Philippines

**Keywords:** genotype shifts, genotype variations, influenza‐like illness, RSV‐A, RSV‐B, severe acute respiratory infection

## Abstract

**Objective:**

Respiratory syncytial virus (RSV) is a leading cause of severe lower respiratory infection, and therefore, a major threat to global health. This study determined the epidemiological and molecular characteristics of RSV among cases of influenza‐like illness (ILI) and severe acute respiratory infection (SARI) among children in the Philippines.

**Method:**

The study included archived nasopharyngeal swab and oropharyngeal swab samples collected from patients under the age of five who are presented with ILI or SARI for the period of 2006–2016. Swabs were examined for RSV subgroup by multiplex real‐time qRT‐PCR. Partial genome sequencing and phylogenetic analyses of the second hypervariable region (HVR) of the G gene were used to determine the genotype of RSV isolates.

**Results:**

A total of 1036 representative samples from all sites were selected and tested. Of these samples, 122 were RSV‐positive at 11.8% prevalence rate, and 58.2% (71/122) were classified as RSV‐A. Six genotypes were identified, which include NA1 (27/122, 22.1%), ON1 (5/122, 4.1%), GA2 (1/122, 0.8%), and GA5 (1/122, 0.8%) for RSV‐A; and BA2 (13/122, 10.7%) and BA9 (1/122, 0.8%) for RSV‐B. Most RSV‐related cases were significantly associated with clinical characteristics such as runny nose (88.1% RSV vs. 11.9% non‐RSV: *p* value = 0.021), pneumonia (80.6% RSV vs. 19.4% non‐RSV; *p* value = 0.015), and bronchitis (71.7% RSV vs. 28.3% non‐RSV; *p* value < 0.001). Increased RSV‐related cases were observed among children below 24 months old.

**Conclusion:**

The RSV trend and genetic variability in the Philippines resembles a similar pattern of transmission globally.

## 1 BACKGROUND

Respiratory syncytial virus (RSV) is a leading cause of severe lower respiratory infections (LRTI) with nearly all children experiencing at least one infection by the age of 2.^1^, ^2^ It is a major health threat that causes about 2,350/100,000 hospitalization among children less than 1 year of age in the United States.^3^

Clinical symptoms of RSV infection among children include prominent wheezing, severe bronchiolitis, cough, and shortness of breath.^4^ It has been known that prematurely born infants, children with congenital heart defects or bronchopulmonary dysplasia, elderly, and immunosuppressed patients have higher risk of developing RSV infections.^5^ Meanwhile, another report showed that 73% of children hospitalized with RSV have no underlying medical conditions.^6^

RSV classification varies antigenically and genetically. Based on the reaction of the monoclonal antibody to the surface antigen, RSV can be divided into two major subgroups: RSV‐A and RSV‐B.^7^ Globally, molecular analysis showed that RSV‐A has 11 known genotypes (GA1–GA7, SAA1, CB‐A, NA1‐4, and ON1), whereas RSV‐B has 24 known genotypes (GB1–GB4, SAB1–SAB4, URU1‐2, CBB, CB1, GB5, and BA1–12).^8^

The Philippines is a subtropical country composed of 17 administrative regions. In the past decades, there were two national disease surveillance systems that were established in the Philippine National Influenza Center in the Research Institute for Tropical Medicine (PNIC‐RITM) to describe the locally circulating patterns of influenza viruses. The influenza‐like illness (ILI) surveillance was initially established in 2006, and the severe acute respiratory infection (SARI) surveillance was later established in 2015. The case definition for ILI cases was based on that of the World Health Organization (WHO), which is defined as patients with acute respiratory illness with cough, runny nose and/or sore throat with history of fever (38°C or above) with or without other system manifestations within the past 5 days. Meanwhile, the Department of Health (DOH) in the Philippines adapted the WHO case definition of SARIwhich is described as patients with an acute respiratory illness that fits the ILI case definition and requires hospitalization. Specifically, the integrated management of childhood illness (IMCI) guidelines also included in the SARI surveillance case definition, any child between 2 months to 5 years of age that are suspect case for pneumonia with cough or difficulty of breathing. Any child between 2 months and 5 years of age with danger signs including inability to drink or breastfeed, vomits everything, convulsion, lethargic, or unconscious, and with chest indrawing or stridor in calm child is classified as severe pneumonia.

Published studies reported RSV as the second most prevalent respiratory viral pathogen next to humanrhinoviruses (HRV) among children with severe pneumonia in the Philippines.^9^ In another study, aside from influenza viruses, RSV (11.0%) and HRV (5.7%) were predominant among ILI cases of adults and children in Leyte Island in the Philippines.^10^ Even though studies have been conducted to describe epidemiology and circulation of genotypes in the Philippines between 2008 and 2015, data prior to this study period have yet been reported on a nationwide scale.

This study examined the epidemiological features of children with RSV‐related infection and characterized the RSV isolates by molecular techniques to identify the prevalence and locally circulating genotypes from 2006 to 2016 in the different regions of the Philippines.

## 2 STUDY DESIGN

### 2.1 Ethical consideration

The study was approved by the Institutional Review Board (IRB) of RITM. Individual consent forms were waived because archived samples were used in this study. A permission letter was sought from the PNIC‐RITM to access the samples and data, respectively. The samples were devoid of any identifiers that may lead to the patient.

### 2.2 Study samples

**FIGURE 1:**
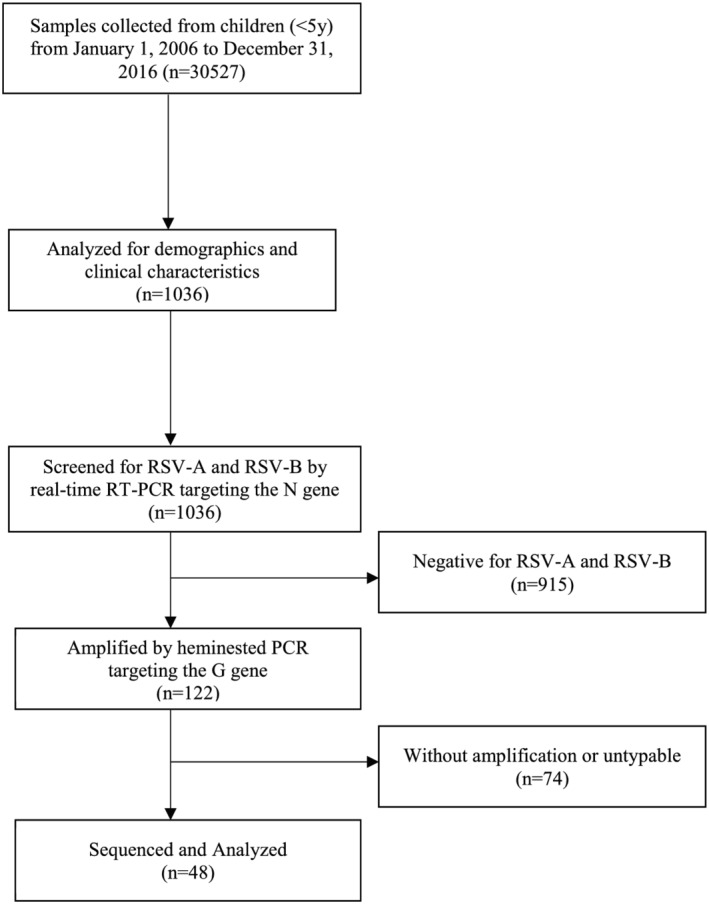
Respiratory syncytial virus (RSV) study flowchart

The study included archived nasopharyngeal swab (NPS) and oropharyngeal swab (OPS) samples of the RITM‐PNIC in Muntinlupa City, Philippines. The number of samples included in the analysis was determined with the aim to calculate the prevalence with specified confidence level of 95% and precision estimate of 3% using the reported prevalence data of 40% in a study on children in Biliran Province in the Philippines.^11^ To achieve a representation of the surveillance samples, then from January 2006 to December 2016, selection was done using systematic sampling for each facility per year of collection. The sampling allocation per facility and year was proportional to the total number of banked samples from children aged 5 years and below with ILI or SARI in each of the facility for every year of collection. The sentinel sites were composed of different 17 hospitals and 34 health centers nationwide that were chosen based on their geographic locations and their capacity to qualify based on the criteria set by the DOH Philippines. Samples were placed in a viral transport medium (VTM) or universal transport medium (UTM) and stored at the institutional biobank (−80°C). Please refer to Figure 1 for the workflow of the study.

### 2.3 Nucleic acid extraction

Viral RNA was extracted from 200 μl of each specimen using Roche High Pure Viral RNA (Roche Applied Sciences, Manheim, Germany) kit based on manufacturer's instructions. RNA was eluted in 50 μl of elution buffer and was stored at −20°C prior to RSV screening.^12^ cDNA was synthesized using primed viral RNA, 5× first strand buffer, 10‐mM DTT, one unit of RNAseOUT, and five units of M‐MLV RT (Moloney Murine Leukemia Virus Reverse Transcriptase, Invitrogen, Carlsbad, CA, USA) for genotype identification.

### 2.4 Virus detection

For RSV screening, multiplex real‐time qRT‐PCR was performed with subgroup‐specific primers and probes used to amplify the partial nucleoprotein or N gene. For genotype identification of all the RSV‐positive samples, the second hypervariable region (HVR) of the glycoprotein (G) gene was amplified by heminested PCR.

### 2.5 Sequencing and phylogenetic analysis

All amplicons were purified using QIAquick Purification Kit (Qiagen, Germany) according to manufacturer's instructions. Only PCR products with a concentration of ≥20 ng/μl were sent to 1st BASE Laboratories in Singapore for Sanger dideoxy sequencing. Resulting sequences from forward and reverse primers were assembled to generate consensus sequences using MEGA 6.^13^ Consensus sequences were aligned using MAFFT^14^ utilizing default parameters. Resulting alignment was manually inspected and confirmed using Aliview.^15^ Appropriate model of nucleotide substitution for the dataset was chosen using jModelTest.^16^ Phylogenetic analysis was performed using maximum‐likelihood method with 1000 bootstrap replicates carried out in IQ‐TREE^17^ and resulting phylogenetic tree was visualized using FigTree v.1.4.0^18^ and Interactive Tree of Life v.5.^19^

### 2.6 Statistical analysis

Data were extracted from the PNIC database. Data were processed and analyzed using STATA 15 (STATA Corporation, College Station, TX, USA) and were presented as counts and proportions. Chi‐square test or Fisher's exact test were used to assess association. The results with *p* value of <0.05 were considered significant.

## 3 RESULTS

### 3.1 Patient characteristics

Between January 1, 2006 to December 31, 2016, a total of 30,527 OPS and NPS samples were collected from children below 5 years old at the ILI or SARI surveillance sentinel sites of the DOH Philippines. Overall, 1,036 samples were systematically chosen, and of these 98.9% (1,024/1,036) were ILI cases and 1.1% (12/1,036) were SARI cases. RSV infection was detected in 11.8% (122/1,036) of the sample population.

Demographic characteristics of RSV (122) and non‐RSV‐related (914) are compared in Table 1. Among male and female RSV and non‐RSV‐related cases, no significant differences were observed. RSV‐related cases were observed to be increased during the first 2 years of life (11.2% to 14.8%) (<24 months).

**TABLE 1:** Demographics and clinical characteristics of RSV cases among children (<5 years) with ILI and SARI in the Philippines, 2006–2016 (n = 1,036)

	RSV PCR result				
	RSV‐negative		RSV‐positive		
	No.	%	No.	%	
	(n = 914)		(n = 122)		*p* value
Sex (n = 1036)					
Male	451	88.1	61	11.9	0.892
Female	463	88.4	61	11.6	
Age group (n = 1036)					
<6 months	111	88.8	14	11.2	0.327
6 to <12 months	178	85.2	31	14.8	
12 to <24 months	248	87.9	34	12.1	
2 to <3 years	162	88.5	21	11.5	
3 to <4 years	115	87.8	16	12.2	
4 to <5 years	100	94.3	6	5.7	
Respiratory symptoms					
Fever (n = 1036)	913	88.2	122	11.8	0.882^a^
Cough (n = 1036)	904	88.1	122	11.9	0.284^a^
Runny nose (n = 1009)	827	88.1	112	11.9	0.021*
Sore throat (n = 674)	162	93.1	12	6.9	0.314
Difficulty of breathing (n = 1015)	123	86.0	20	14.0	0.321
Crackles (n = 1016)	11	91.7	1	8.3	0.594^a^
Tonsilitis (n = 1019)	13	86.7	2	13.3	0.524^a^
Respiratory wheezing (n = 737)	33	91.7	3	8.3	0.312^a^
Diagnosis					
Asthma (n = 768)	17	81.0	4	19.0	0.288^a^
Bronchitis (n = 747)	38	71.7	15	28.3	<0.001*
Pneumonia (n = 760)	125	80.6	30	19.4	0.015*

*Note*: Chi‐square test used unless otherwise mentioned.

^a^ Fisher's exact test was used,

* *p* value of <0.05 was considered significant.

### 3.2 Clinical characteristics of RSV infection

Runny nose (88.1% RSV vs. 11.9% non‐RSV: *p* value: 0.021) was significantly more common among RSV‐positive cases. Moreover, most RSV‐positive cases were significantly associated with pneumonia (80.6% RSV vs. 19.4% non‐RSV; *p* value: 0.015) and bronchitis (71.7% RSV vs. 28.3% non‐RSV; *p* value: <0.001).

### 3.3 RSV distribution

**FIGURE 2:**
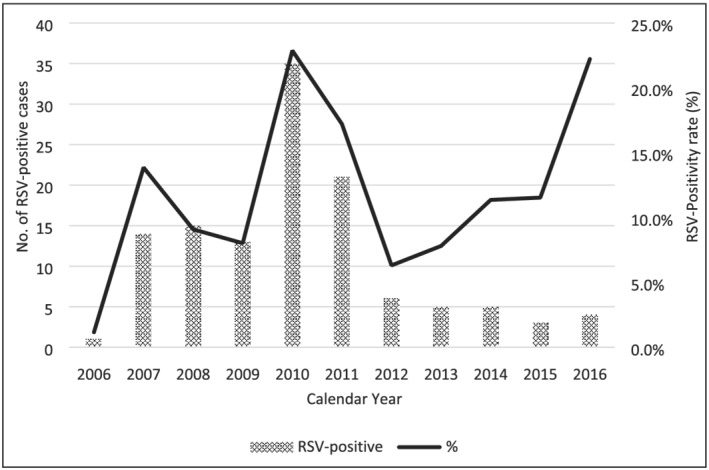
Temporal distribution of RSV‐related ILI and SARI cases of children (<5 years) in the Philippines, 2006–2016

Annual positivity rates of RSV infection varied across the study period as seen in Figure 2. Positivity rates ranged from 1.2% (2006) to 22.9% (2010) with an overall prevalence rate of 11.8% (122/1,036). The highest positivity rates were observed in 2010 and 2011, respectively. RSV infection was observed all throughout the year, however increased positivity rate among specimens collected during the second half of the year (June to December) was documented. The study showed that 58.2% (71/122) of the RSV‐positive specimens belonged to the subgroup RSV‐A while 40.1% (49/122) to RSV‐B. Coinfection between both RSV subgroups was observed in 1.6% (2/122) of the samples. Cocirculation between both subgroups was observed for the period of 2007–2015, with higher number of RSV‐A related cases (50.0%–100.0%).

### 3.4 Phylogenetic analysis of RSV

Among the confirmed RSV samples, only 48 were amplified during the heminested PCR targeting the second HVR of the G protein. The remaining 74 RSV isolates (37 RSV‐A, 35 RSV‐B, and 2 coinfection) that failed to amplify were not included for sequencing and phylogenetic analysis, and were classified as untypable. All sequences generated from this study were deposited in GenBank under accession nos. OM962798–OM962831 for RSV‐A and OM962832–OM962845 for RSV‐B.

**FIGURE 3:**
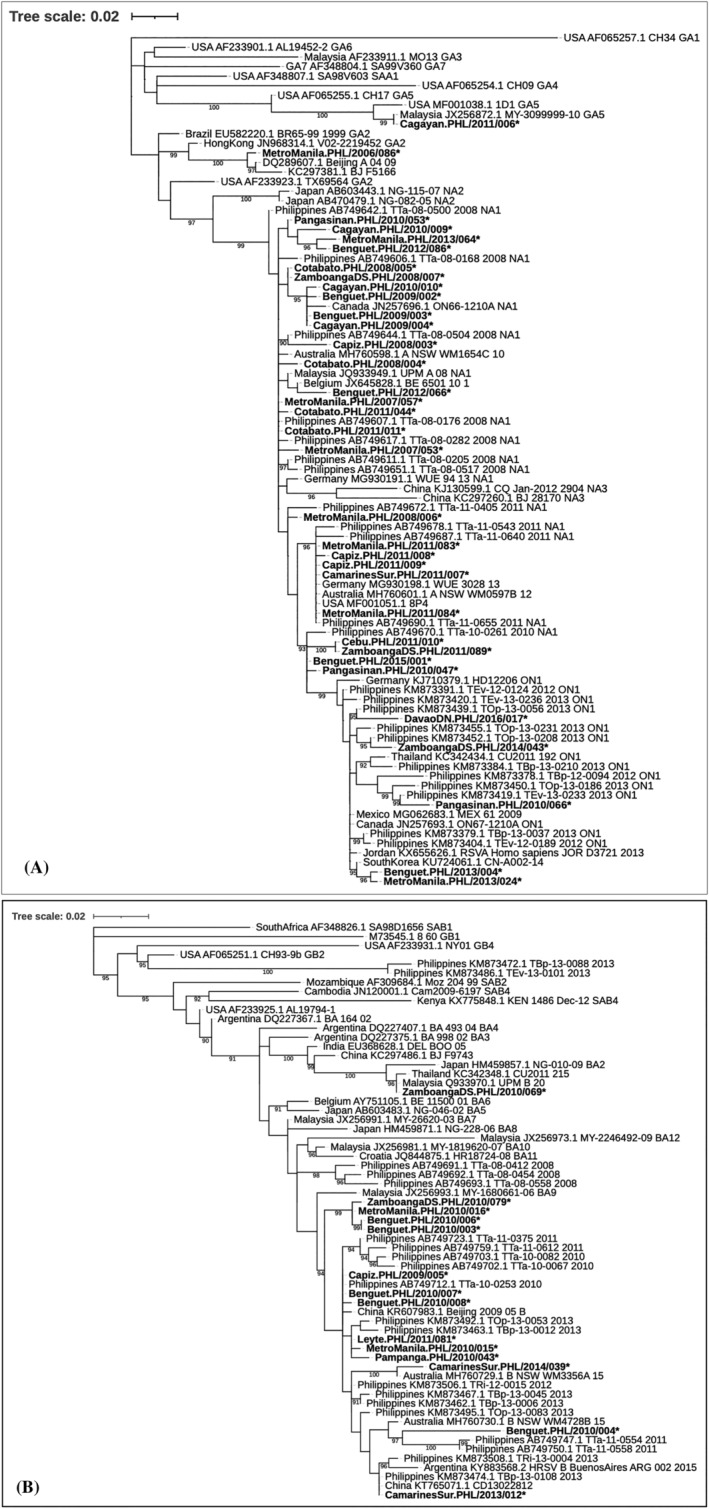
Phylogenetic trees of (A) RSV‐A and RSV‐B (B) partial G gene region. The trees were inferred from the partial G gene regions by the maximum‐likelihood method, using bootstrap values calculated from 1000 trees. Sequences about 333 bp for RSV‐A and 324 bp for RSV‐B were included in the analysis. Only bootstrap values higher than 90% were shown. Strains isolated in the Philippines for this study are boldfaced and marked with asterisks

Phylogenetic trees were constructed for 34 RSV‐A and 14 RSV‐B strains by including 45 (23 RSV‐A and 20 RSV‐B) Philippine strains from previous studies^20^ and 97 (46 RSV‐A and 51 RSV‐B) from other countries by BLASTn search (Figure 3A,B). Philippine RSV‐A strains in this study were distributed into four clusters NA1, ON1, GA2, and GA5. Philippine NA1 and ON1 strains clustered with previously reported circulating strains in the Philippines and other countries. Philippine GA2 strains clustered with strains from Brazil and HongKong. Meanwhile, the Philippine GA5 strain clustered with strains from Malaysia and USA. Majority of the Philippine RSV‐A strains were identified as NA1 genotype (27/122, 22.1%). Respectively, the following RSV‐A genotypes were identified: ON1 (5/122, 4.1%), GA2 (2/122, 1.6%), and GA5 (1/122, 0.8%).

All Philippine RSV‐B strains in this study clustered under the BA strains with 60‐nucleotide duplication. Most of the Philippine BA strains were identified as BA9 genotype (13/122, 10.6%) except for one (1/122, 0.8%) that was identified as BA2. Philippine BA2 strains clustered under strains from Japan, Thailand, and Malaysia while Philippine BA9 strains under strains from Malaysia and Colombia.

### 3.5 Prevailing RSV genotype shifts

**FIGURE 4:**
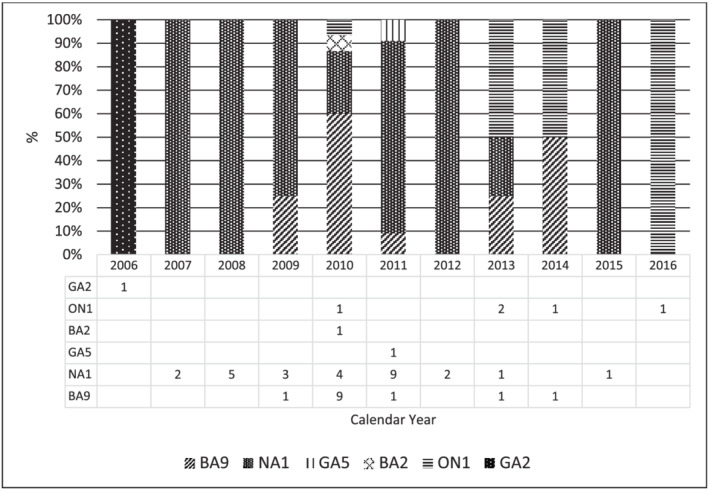
Temporal distribution of RSV‐related ILI and SARI cases of children (<5 years) in the Philippines by genotype, 2006–2016 (n = 48). NA1 is the predominant genotype between 2007 and 2012. BA9 was only predominant in 2010

Genotype shift among the circulating RSV‐A and RSV‐B genotypes was observed within the 10‐year study period as seen in Figure 4. NA1 genotype was detected as early as February 2007 in the Metro Manila, Philippines, and continued to circulate until 2015. BA9 genotype was detected in the in the province of Capiz located in the western region of the Visayas islands in the Philippines in 2009 until 2013. This study was able to detect ON1 genotype in the province of Pangasinan, which is in the western area of Luzon as early as June 2010. Simultaneous detection of genotypes BA2 in the province of Zamboanga del Sur in Mindanao and BA9 (Cagayan, Philippines) were reported within the same year.

## 4 DISCUSSION

To our knowledge, this investigation is the first study to provide long‐term Philippine data on the epidemiology and molecular characteristics of circulating RSV among children (<5 years) who presented with either ILI or SARI over a period of 10 years (2006–2016). We described the genetic variability of RSV isolates in the country, together with the reported cocirculation of both RSV‐A and RSV‐B subgroups. We observed an increased detection of RSV‐A subgroup all throughout the study period, except for 2010. It must be noted that the different time interval in the shift of dominance among RSV subgroups had been previously described. A study from the South Western China showed a different predominance pattern, with RSV‐A prevailing in the period of 2006–2008 and RSV‐B prevailing during the subsequent season (2008–2009).^21^ Furthermore, we described the clustering of the Philippine RSV‐A strains into four genotypes (GA2, GA5, NA1, and ON1) while RSV‐B strains into two genotypes (BA2 and BA9). These findings were also analyzed with the available demographics and clinical characteristics of the patients in the study.

The present study described a broader and more robust picture of the shifting of RSV genotypes in the Philippines from 2006 to 2016. This is in comparison with previous studies in the country, which covered shorter study periods and a different timeframe from 2008 to 2012^20^ and 2012 to 2015.^22^ For the RSV‐A strains, we discovered a pattern of shifting among genotypes GA2, GA5, NA1, and ON1 based on the time of first detection in our country, and we report the circulation of the only Philippine GA2 strain in 2006, which was detected earlier than the only reported Philippine GA5 strain in 2010. This finding is different compared with previous reports in the United States, which showed the predominance of GA5 during the period of 2004 to 2006, which was then replaced by GA2 in 2007^23^; and another study in Taiwan which reported GA2 becoming the predominant genotype after 2005.^24^ The detection of the only Philippine GA5 strain in 2010 is consistent with the reported occasional detection of GA5 in Spain.^25^ We also observed a gradual replacement of the prevailing strain from GA2 to NA1 and from NA1 to ON1. Our data showed the first Philippine NA1 strain was detected in 2007 and continued to circulate until 2015. With this, we also observed cocirculation of genotypes NA1 and GA5 in 2010, which is similar with a report in India in 2012,^26^ whereas the first detection of Philippine ON1 strain appeared in 2010 and continued to prevail until 2016. Our findings suggest that both genotypes were already circulating in the Philippines a year earlier than the previously reported first detection of Philippine NA1 in 2008^20^ and Philippine ON1 in 2011.^22^

Since the first identification of the GA2 genotype in 1998, GA2 branched into two clusters, namely, NA1 and NA2, which then on NA1 became the prevailing genotype worldwide until it was subsequently replaced by ON1 between 2009 and 2010.^27^, ^28^, ^29^, ^30^, ^31^ For the RSV‐B strains, we only reported genotypes BA2 and BA9 circulation. We reported the detection of the Philippine BA9 genotype in 2009 and predominance in 2010. This is consistent with the previous reports that described the emergence and predominance of BA9 among children in the Philippines from 2009 to 2011.^22^, ^32^ Taking all the data from related literatures and the present finding, temporal factor plays a big role in the variation of RSV genotypes worldwide. With this, the shifting pattern of RSV genotypes in the Philippines reflects similarly the global RSV activity. We also suggest that the possible replacement of older RSV genotypes with the new genotypes could be due to the increasing herd immunity of the hosts against the previously circulating genotypes. Data from this study can be utilized in the integration of the monitoring of RSV activity in the national surveillance systems in the country.

The reported overall RSV prevalence rate in this study (11.8%) is consistent with published rates among children (<5 years) with ILI among nearby countries such as Thailand (13.2%) within the period of 2012–2018^33^ and Singapore (9.4%) from 2014 to 2018.^34^ Because the data for this study are mostly from ILI cases (98.9%), there is a difference compared with the prevalence rates in other published articles in the country. Previous studies in the Philippines reported a higher range of prevalence rate between 19.3% and 40.6%. It must be noted that these studies were conducted among children hospitalized for pneumonia in Biliran Province, Palawan, and Baguio City in the Philippines.^11^, ^20^, ^22^, ^35^ Therefore, we suggest that RSV is more prevalent among hospitalized children. With this, we also investigated the different host factors and clinical characteristics that are related to RSV infection.

RSV‐related cases were mostly observed among children below 24 months old or below 2 years of age. This is reflected in similar studies in Singapore where age‐specific RSV positivity was observed in infants and toddlers (≤2 years).^34^ With this, we also reported runny nose, pneumonia, and bronchitis as clinical characteristics significantly related to RSV infection. This agrees with other published reports that referred RSV as the main agent responsible for pneumonia during the first year of life and main cause of bronchiolitis among hospitalized patients.^36^ A study conducted in the Philippines among children with cases of severe pneumonia reported increased viral load among infants (<6 months), which was associated with the NA1 genotype.^11^ These data could also be due to host‐related factors such as differing airway anatomy, immaturity of the immune system, and waning levels of maternal antibodies, which could have contributed to the vulnerability of younger children to severe infections.^37^ Since currently there are no commercially available vaccines for this age group,^38^ our finding reiterate the importance of fast tracking the development of an effective vaccine against RSV for younger children.

Although RSV‐related cases are present all throughout the year, our data showed that higher positivity rates can be observed in the second half of the year (June to December). Because thePhilippines is a subtropical country, these months are commonly identified with heavy rainfall and increased number of tropical cyclones.^2^ This finding is similar with a study in Biliran Province in the Philippines, which reported two RSV epidemics, with the first epidemic occurring between May 2014 and January 2015 and the second between October 2015 and January 2016.^39^ It must be noted that the Philippines is an archipelago country, and therefore, difference in the climates within the different islands and regions can potentially be observed. One of the strengths of this present study is that we utilized the national ILI and SARI surveillance samples that allowed a more comprehensive data source. Although there were varying number of outpatient healthcare centers and hospitals yearly due to administrative and financial factors, these areas were strategically chosen to represent the different major regions in the Philippines. Other Southeast Asian countries that share the same tropical and subtropical climate including Cambodia,^40^ Vietnam,^35^, ^41^ and Singapore^34^ compliment our data. With this, our finding could be a reference for future detailed seasonality studies to determine the different climactic factors affecting the behavior of RSV in the Philippines.

Several limitations were identified in this study. First major limitation is the retrospective aspect of the study that utilized frozen archived samples from the ILI and SARI surveillances from 2006 to 2016. These samples were primarily used for the detection of influenza viruses by virus isolation and factors such as frequent freeze thawing, difference in sample collection procedures, and probable poor specimen conditions during transport from different geographical location of the sentinel sites could have affected the quality of RNA in the samples tested. These factors are suggested to have led to a large percentage (60.7%) of RSV‐A and RSV‐B isolates that were not genotyped or classified as “untypable” due to low viral concentration. Due to lack of representative RSV genotypes per year, the specific period for the displacement of RSV genotypes in the country cannot be fully described. Association of clinical characteristics in relation to specific genotypes was not included in the analysis. Another limitation is the varying sources of the samples that utilized SARI and ILI case definitions. Currently, the WHO established a Global RSV Surveillance Extension Phase (Phase II) that recommended the use of the extended SARI case definition. This case definition is more specific for RSV monitoring with extended SARI cases defined as severe (overnight, or more than 24 h of hospital admission), acute and respiratory infection (cough or shortness of breath). In infants less than 6 months of age, symptoms also include apnea or sepsis (fever, shock, and serious illness without apparent cause).^42^ With this, suspect RSV cases do not require fever presentation to be admitted in the RSV surveillance. It should be noted that a considerable fraction (>50%) of RSV‐infected young children and elderly patients present without fever in the RSV surveillance. In line with this, another limitation is the fewer number of SARI (1.1%) in comparison with ILI samples (98.9%) included in the study. The national ILI surveillance in the Philippines was established in 2010, whereas the national SARI surveillance was only started in 2015. With fewer SARI representative samples for 2015 and 2016, the severity and burden of hospitalization of RSV was not fully investigated in this study.

In conclusion, this study showed that RSV is more prevalent among younger children (<2 years) in the Philippines. In addition, runny nose, bronchitis, and pneumonia are clinical manifestations related to RSV infections. The study also identified cocirculation of both RSV subgroups (RSV‐A and RSV‐B) and shifting of RSV genotypes (GA2, GA5, NA1, and ON1 for RSV‐A and BA2 and BA9 for RSV‐B) within the study period. This work is the first study to report, on a nationwide scale, the prevalence of RSV and shift of prevailing RSV genotypes over the period of 2006–2016. The pattern of RSV activity in the Philippines resembles global transmission patterns. Data from this study can be utilized to aid policy makers in the country to recognize the importance of continuous monitoring of RSV activity and integrate it in national surveillance systems.

## AUTHOR CONTRIBUTIONS

**Jonjee Morin:** Conceptualization; data curation; formal analysis; funding acquisition; investigation; methodology; project administration; resources. **Vina Lea Arguelles:** Conceptualization; supervision. **Janiza Lianne Foronda:** Formal analysis; methodology; validation; visualization. **Alvin Tan:** Formal analysis. **Socorro Lupisan:** Funding acquisition; resources.

### PEER REVIEW

The peer review history for this article is available at https://publons.com/publon/10.1111/irv.12986.

## Data Availability

The data that support the findings of this study are available from the corresponding author upon reasonable request.
